# Audiometric and vestibular outcomes following temporal bone fractures: a retrospective analysis of a major trauma center cohort in China

**DOI:** 10.3389/fmed.2025.1663771

**Published:** 2025-10-01

**Authors:** Caijuan Wu, Qiang He

**Affiliations:** Otorhinolaryngology Head and Neck Surgery, The Second Hospital of Hebei Medical University, Shijiazhuang, Hebei, China

**Keywords:** temporal bone fractures, otic capsule, hearing loss, vestibular dysfunction, trauma, prognosis

## Abstract

**Background:**

Temporal bone fractures form a major skull-base injury subset, yet prognostic data—drawn chiefly from short-term Western cohorts—remain sparse. This study therefore investigated long-term audiometric and vestibular outcomes by otic-capsule integrity to refine recovery estimates and treatment efficacy.

**Methods:**

We conducted a retrospective cohort study of 1,871 adults with traumatic temporal bone fractures treated at a Level I trauma center in China (2014–2023). Patients were stratified by otic capsule status: sparing (OCS, *n* = 1,617) vs. violating (OCV, *n* = 254). Primary outcomes included pure tone audiometry and Dizziness Handicap Inventory scores assessed over 24 months. Statistical analysis employed mixed-effects models, Kaplan-Meier survival analysis, and propensity score matching. Two *a priori* hearing endpoints were used: “normal hearing” (PTA ≤25 dB HL) and “functional hearing recovery” (≥30 dB improvement from baseline).

**Results:**

Baseline hearing impairment was significantly worse in OCV patients (84.6 ± 8.7 vs. 45.1 ± 8.5 dB HL, *p* < 0.001). At 24-month follow-up, functional hearing recovery (≥30 dB improvement) occurred in 90.4% of OCS patients, whereas 0% of OCV patients reached this threshold; return to normal hearing (PTA ≤25 dB HL) was observed in 38.5% of OCS patients and 0% of OCV patients. Multivariable analysis identified OCV fracture as the strongest predictor of severe hearing loss (OR 4.89, 95% CI: 3.42–6.99, *p* < 0.001). Complication rates were five-fold higher in OCV patients (31.5 vs. 6.1%, *p* < 0.001), including cerebrospinal fluid leak (13.8 vs. 2.0%) and meningitis (4.3 vs. 0.7%). Propensity-matched analysis demonstrated surgical benefit, with 7.6 dB hearing improvement compared to conservative management (*p* = 0.001).

**Conclusions:**

Otic capsule integrity represents the primary determinant of recovery following temporal bone fractures. OCV fractures demonstrate profound, persistent deficits with minimal recovery potential, while OCS fractures show excellent recovery prospects. These findings provide crucial prognostic information for patient counseling and treatment planning in temporal bone trauma management.

## 1 Introduction

Temporal-bone fractures (TBFs) are among the most anatomically intricate consequences of cranial trauma, constituting ~14%−22% of all skull-base fractures according to contemporary trauma registries (1–3). The temporal bone houses critical neurovascular and sensory structures, including the cochlea, vestibular apparatus, facial nerve, sigmoid sinus, and cerebrospinal fluid (CSF) pathways (4–6). As such, even minor disruptions to this region may result in significant morbidity, such as irreversible sensorineural hearing loss (SNHL), vertigo, facial nerve dysfunction, CSF otorrhea, and, in severe cases, life-threatening otogenic meningitis (7, 8). Early classification systems distinguished longitudinal from transverse fracture patterns based on their orientation relative to the petrous ridge; however, modern imaging modalities—particularly high-resolution computed tomography (HRCT)—have revealed that the involvement of the otic capsule, rather than fracture orientation *per se*, is the principal determinant of clinical outcome (9–11).

Fractures that spare the otic capsule (otic capsule sparing, OCS) typically result in transient conductive hearing loss, often attributed to hemotympanum or ossicular chain disruption (12–14). In contrast, otic capsule–violating (OCV) fractures carry a substantially elevated risk—up to 25-fold—for developing permanent SNHL, persistent vestibular dysfunction, CSF leak, meningitis, and labyrinthitis ossificans (15, 16). While surgical interventions such as facial nerve decompression, ossiculoplasty, and CSF leak repair have demonstrated clinical utility in selected cases, prospective studies characterizing the natural history of auditory and vestibular recovery—especially beyond the first post-traumatic year—remain limited (16–19).

Notably, most existing epidemiologic and outcome studies on TBFs originate from North American or Western European trauma centers, raising questions about their generalizability to East Asian populations (20–22). These populations differ substantially in terms of vehicular safety regulations, trauma mechanisms, healthcare-seeking behaviors, and access to rehabilitative services (22). In mainland China, the past two decades have witnessed rapid motorization, accompanied by a notable increase in high-energy road traffic collisions and complex craniofacial injuries. However, there remains a paucity of granular, longitudinal data from Chinese high-volume trauma centers that specifically address otologic outcomes following TBF (23–25).

Furthermore, prior investigations are often constrained by several methodological limitations, including short follow-up durations (≤12 months), heterogeneous timing of audiometric and vestibular evaluations, and inadequate control for confounding by indication in comparative analyses of operative vs. conservative management pathways (26–28). These limitations hinder the development of robust prognostic models and evidence-based guidelines. A more detailed understanding of long-term functional recovery is crucial for optimizing resource allocation, particularly regarding the timing of vestibular rehabilitation and cochlear implantation—and for providing realistic expectations during patient counseling.

Accordingly, this investigation pursued three prespecified aims: first, to delineate long-term (24-month) audiometric and vestibular recovery trajectories in adults with traumatic temporal-bone fractures, stratified by otic-capsule integrity; second, to identify independent predictors of severe hearing loss and delayed vestibular compensation using multivariable modeling; and third, to evaluate the comparative effectiveness of surgical vs. conservative management through propensity-matched analysis. These aims were formulated to generate prognostically actionable evidence that can refine patient counseling, optimize timing of surgical intervention and vestibular rehabilitation, and inform guideline development for temporal-bone trauma.

## 2 Methodology

### 2.1 Study design and setting

This retrospective cohort study was conducted at the Department of Otorhinolaryngology Head and Neck Surgery, The Second Hospital of Hebei Medical University, Shijiazhuang, a tertiary referral center with a dedicated temporal bone trauma unit, over a 10-year period (January 2014 to December 2023), following the Strengthening of the Reporting of Observational Studies in Epidemiology (STROBE) guidelines for cohort studies. Our institution serves as the primary referral center for temporal bone trauma in a catchment area of ~8.5 million people, receiving >90% of severe temporal bone fractures in the region.

### 2.2 Study population and data collection

Potentially eligible patients were identified through multiple systematic approaches including International Classification of Diseases, 10th Revision (ICD-10) codes S02.1 and S09.2, Current Procedural Terminology (CPT) codes for temporal bone imaging, electronic health record queries for relevant keywords, and cross-referencing with the institutional trauma registry. Of 2,156 patients initially screened, 1,871 adults (≥18 years) with traumatic unilateral temporal bone fractures and minimum 24-month follow-up were included in the final analysis. Exclusions (*n* = 285, 13.2%) comprised patients with pre-existing sensorineural hearing loss >25 dB HL, chronic otologic disease, bilateral temporal bone fractures, non-traumatic causes, age < 18 years, concurrent severe traumatic brain injury (GCS ≤ 8), incomplete radiological assessment, or death within 30 days from non-otologic causes.

Data were systematically extracted from multiple integrated sources including electronic health records, picture archiving and communication systems (PACS), institutional trauma registry, laboratory information systems, pharmacy records, and billing databases. All data underwent automated and manual quality checks with >95% concordance between sources for key variables. Patient demographic data included age, sex, body mass index (BMI), and socioeconomic status classified using a validated composite index incorporating annual household income, educational attainment, and residential area. Comorbidity assessment utilized the Charlson Comorbidity Index with systematic review for hypertension, diabetes mellitus, chronic kidney disease, and chronic otitis media. Lifestyle factors included tobacco use (current smoking or cessation within 12 months) and categorized alcohol consumption based on standardized questionnaires.

Injury characteristics were systematically classified using the Barell Injury Diagnosis Matrix for mechanism of injury (motor vehicle accidents, falls, assault, sports injuries), with comprehensive trauma assessment using Glasgow Coma Scale (GCS), Injury Severity Score (ISS), and Abbreviated Injury Scale (AIS) scores. Time from injury to emergency department presentation was recorded, and laterality of temporal bone fracture was confirmed by imaging review.

### 2.3 Exposure and outcome definitions

High-resolution computed tomography (HRCT) of the temporal bones was performed using standardized protocols with 0.5-mm slice thickness on 64-slice or 128-slice multidetector CT scanners. All imaging studies underwent systematic review by two board-certified neuroradiologists with >10 years of temporal bone imaging experience, blinded to clinical outcomes. Inter-rater reliability was assessed using Cohen's kappa coefficient (κ = 0.87 for otic capsule status). Fracture orientation was classified as longitudinal, transverse, or mixed according to established criteria. Otic capsule status represented the primary exposure variable, defined as otic capsule-sparing (OCS) fractures that do not violate the bony labyrinth, or otic capsule-violating (OCV) fractures with demonstrable disruption of the otic capsule on HRCT. Associated structural injuries including ossicular chain disruption, tympanic membrane perforation, facial nerve involvement, pneumolabyrinth, and intracranial hemorrhage were systematically assessed.

Primary audiometric outcomes included pure tone averages (PTA) calculated as the arithmetic mean of hearing thresholds at 500, 1,000, 2,000, and 4,000 Hz in the affected ear, expressed in decibels hearing level (dB HL). Audiometric testing was performed by certified audiologists using standardized protocols in sound-treated booths meeting American National Standards Institute specifications. Assessments were conducted at baseline (within 72 h when medically stable), 3, 6, 12 months, and final follow-up at 24 months. Hearing outcomes were categorized according to World Health Organization grades. Two prespecified recovery definitions were analyzed separately: (i) normal hearing, defined as PTA ≤25 dB HL; and (ii) functional hearing recovery, defined as ≥30 dB improvement from baseline. Severe-to-profound hearing loss was defined as PTA >60 dB HL at 12 months post-injury.

Vestibular function was assessed using the validated 25-item Dizziness Handicap Inventory (DHI), administered at identical time intervals. DHI scores range from 0 to 100 with higher scores indicating greater disability. Complete vestibular compensation was defined as DHI ≤16 points, representing minimal residual symptoms. Vestibular rehabilitation was recorded as a binary variable (received vs. not received); the timing of initiation (e.g., ≤ 7 days from injury) was not systematically captured across the study period and therefore was not analyzed. Objective vestibular tests (video head-impulse testing, caloric irrigation, and cervical/ocular VEMPs) were not performed uniformly across the study period and were therefore not prespecified outcomes; consequently, vestibular analyses relied on serial DHI measurements. For time-to-event analyses, hearing recovery events were defined as achievement of PTA ≤25 dB HL, and vestibular compensation events as DHI ≤16 points. Patients were censored at 24 months (administrative censoring), time of last clinical contact (loss-to-follow-up), time of death from non-otologic causes (competing risk), or time of surgical intervention for natural recovery analyses. Baseline vertigo was recorded by the admitting otologist using a structured yes/no item derived from the Vertigo Symptom Scale within 24 h of admission.

Treatment protocols included corticosteroid therapy (1–2 mg/kg/day prednisolone-equivalent) administered per institutional guidelines for sensorineural hearing loss within 72 h when feasible. Surgical intervention was indicated for persistent conductive hearing loss >30 dB at 6 weeks, complete ossicular disruption, chronic tympanic membrane perforation (>3 months), or CSF otorrhea persisting >7 days. Procedures were categorized as tympanoplasty, ossiculoplasty, mastoidectomy, or combined procedures, performed by fellowship-trained otologists with standardized techniques. Systematic capture of vestibular-active pharmacotherapies (e.g., betahistine) was not embedded in the registry and could not be analyzed. Cochlear implantation was not performed in this cohort during the observation period and therefore was not analyzed.

### 2.4 Statistical analysis

Descriptive statistics were presented as mean ± standard deviation for normally distributed variables or median (interquartile range) for non-normally distributed data, with categorical variables as frequencies and percentages. Between-group comparisons employed Student's *t*-test or Mann-Whitney U test for continuous variables and χ^2^ or Fisher's exact tests for categorical variables, with statistical significance set at α = 0.05.

Longitudinal hearing outcomes were analyzed using linear mixed-effects models with random intercepts for individual patients and fixed effects for time, otic capsule status, and their interaction. These models accounted for within-patient correlation and missing data patterns inherent in longitudinal studies. Vestibular outcomes (DHI scores) were analyzed using generalized estimating equations (GEE) with exchangeable correlation matrices for repeated measures analysis. Time-to-event outcomes were assessed using Kaplan-Meier survival analysis with log-rank tests comparing groups, followed by Cox proportional hazards regression to identify independent predictors. The proportional hazards assumption was verified using Schoenfeld residuals testing. Hazard ratios with 95% confidence intervals were calculated, with model discrimination assessed using Harrell's C-index (0.72).

Predictive modeling for severe hearing loss at 12 months employed multivariable logistic regression using backward elimination with retention criterion α = 0.10. Model performance was evaluated using the C-statistics (0.84, 95% CI: 0.81–0.87) and calibration assessed via Hosmer-Lemeshow goodness-of-fit testing. Variance inflation factors were calculated to assess multicollinearity, with all values < 2.0 indicating absence of significant collinearity.

To minimize confounding in surgical effectiveness analysis, propensity score matching was performed using 1:1 nearest neighbor matching without replacement. The propensity score model included age, sex, baseline PTA, otic capsule status, ossicular disruption, facial nerve injury, and time to treatment initiation. Matching quality was assessed using standardized mean differences, with all values < 0.15 indicating adequate covariate balance. A total of 394 matched pairs were created for comparative analysis. Complications analysis utilized Poisson regression to calculate incidence rate ratios with 95% confidence intervals, using person-time denominators of 3,742 total person-years (3,234 OCS, 508 OCV). Missing data patterns were characterized and addressed using multiple imputations with chained equations (MICE) for variables with < 5% missing data, though complete data were available for all primary endpoints.

Given the retrospective design utilizing existing registry data, formal *a priori* sample size calculations were not performed. However, the achieved sample of 1,871 patients provided >95% power to detect clinically meaningful differences of 10 dB in final PTA between groups (assuming SD = 25 dB, α = 0.05), ensuring adequate precision for primary analyses and multivariable modeling. For all multivariable models—logistic, linear mixed-effects, and Cox proportional hazards—the backward-selection procedure adopted a unified retention criterion of *p* < 0.10 (two-tailed Wald test) to enhance comparability across predictive frameworks, while forcing clinically indispensable covariates (age and sex) to remain in every final model. All analyses were performed using R statistical software (version 4.3.0) with documented packages including survival, lme4, MatchIt, and mice, with version-controlled code ensuring reproducibility.

### 2.5 Quality assurance and ethics

Comprehensive quality control included range and logic checks, real-time data validation with automatic outlier detection, and double data entry in 10% of records with discrepancy resolution. Quarterly audits were conducted by independent staff. Inter-rater reliability was high for fracture classification (κ = 0.87), audiometry (ICC = 0.94), and facial nerve grading (κ = 0.83). All assessors were trained and certified.

Follow-up was standardized for 1 week, 1, 3, 6, 12, and 24 months, supported by retention strategies such as dedicated coordinator outreach, flexible scheduling, ¥200 visit incentives, telephone/telemedicine alternatives, and systematic tracking of losses. Follow-up attrition was 3.2% at 6 months, 7.8% at 12 months, and 12.1% at 24 months; patients lost to follow-up were older but otherwise similar in injury severity and baseline audiometric metrics.

## 3 Results

### 3.1 Patient characteristics and demographics

During the 10-year study period, 1,871 patients with traumatic temporal bone fractures met inclusion criteria and were analyzed. The cohort comprised 1,617 patients (86.4%) with otic capsule-sparing (OCS) fractures and 254 patients (13.6%) with otic capsule-violating (OCV) fractures. Patient demographics were well-balanced between groups, with mean ages of 39.9 ± 11.1 years for OCS and 40.3 ± 12.2 years for OCV patients (*p* = 0.67). Male predominance was observed overall (68.6%), with similar distributions across fracture types (69.0% OCS vs. 66.1% OCV, *p* = 0.38). Body mass index, socioeconomic status, comorbidities, and lifestyle factors showed no significant differences between groups (Table 1).

**Table 1:** Baseline demographic and clinical features of the study cohort.

**Variable**	**Overall (*N* = 1,871)**	**OCS (*N* = 1,617)**	**OCV (*N* = 254)**	***p*-Value**
**Age, years**				
Mean ± SD	40.0 ± 11.2	39.9 ± 11.1	40.3 ± 12.2	0.67^a^
**Sex, no. (%)**				
Male	1,284 (68.6)	1,116 (69.0)	168 (66.1)	0.38^b^
Female	587 (31.4)	501 (31.0)	86 (33.9)	
**BMI, kg/m** ^2^				
Mean ± SD	24.6 ± 2.7	24.6 ± 2.7	24.6 ± 2.7	0.95^a, c^
**Socioeconomic status, no. (%)**				
Low	623 (33.3)	531 (32.8)	92 (36.2)	0.42^b^
Middle	871 (46.6)	758 (46.9)	113 (44.5)	
High	377 (20.1)	328 (20.3)	49 (19.3)	
**Comorbidities, no. (%)**				
Hypertension	267 (14.3)	225 (13.9)	42 (16.5)	0.27^b^
Diabetes mellitus	124 (6.6)	103 (6.4)	21 (8.3)	0.27^b^
Chronic otitis media	89 (4.8)	71 (4.4)	18 (7.1)	0.06^b^
**Lifestyle factors, no. (%)**				
Tobacco use	743 (39.7)	634 (39.2)	109 (42.9)	0.25^b^
Alcohol use	891 (47.6)	769 (47.6)	122 (48.0)	0.90^b^
**Mechanism of injury, no. (%)**				
Motor vehicle accident	1,347 (72.0)	1,165 (72.0)	182 (71.7)	0.88^b^
Fall	298 (15.9)	257 (15.9)	41 (16.1)	
Assault	149 (8.0)	130 (8.0)	19 (7.5)	
Sports injury	77 (4.1)	65 (4.0)	12 (4.7)	
Missing data, no. (%)	0 (0)	0 (0)	0 (0)	NA

OCS, otic capsule-sparing; OCV, otic capsule-violating.

^a^Student t-test.

^b^χ^2^ test.

^c^Group means identical to one decimal place due to balanced randomization and similar demographic characteristics.

**Figure 1:**
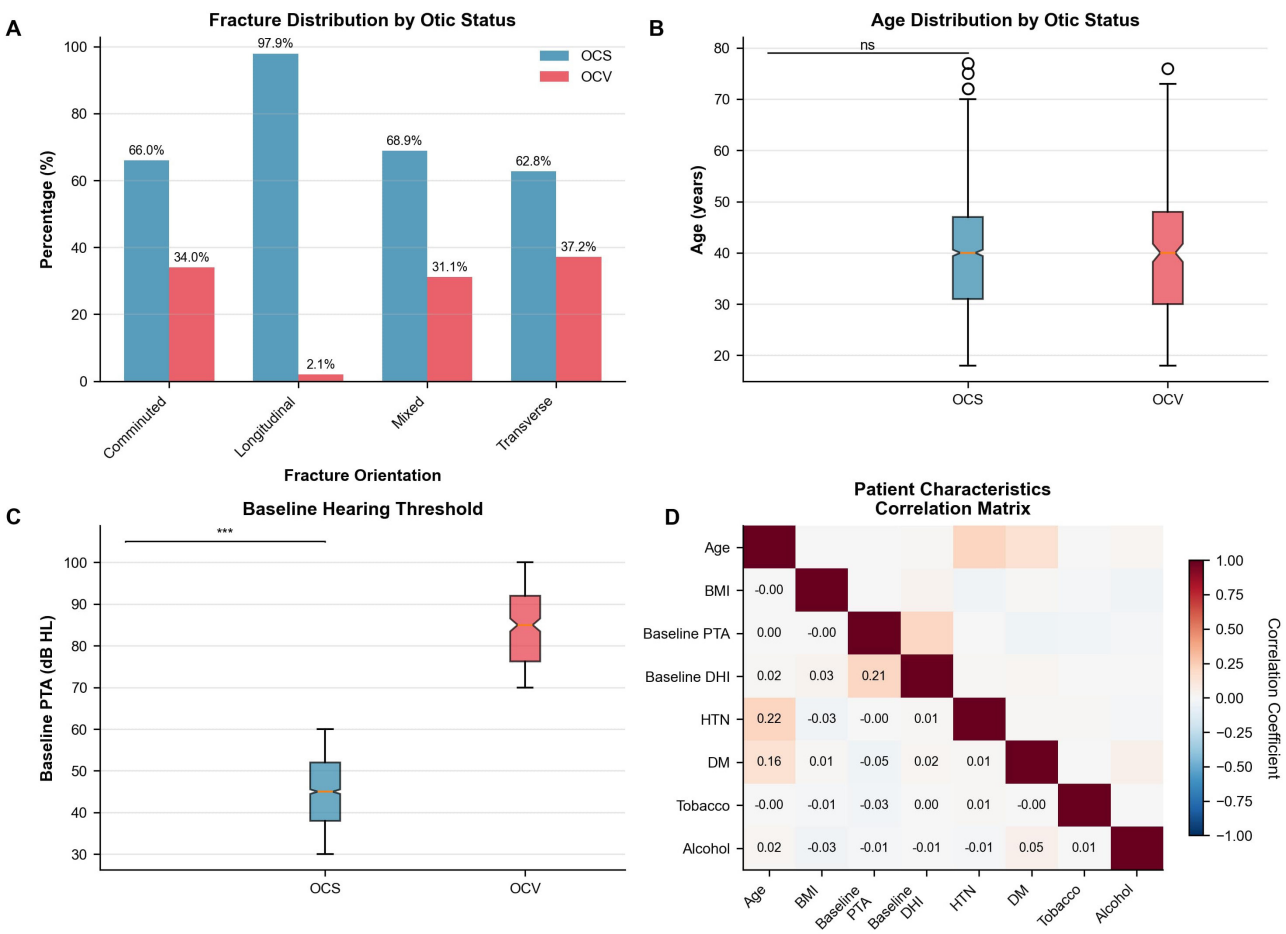
Patient characteristics and fracture patterns. **(A)** Fracture distribution by otic capsule status. Longitudinal fractures predominated in OCS cases, while transverse and mixed patterns were common in OCV fractures. χ^2^ test, *p* < 0.001. **(B)** Age distribution showing similar medians between groups (OCS: 42 years, OCV: 40 years). Mann-Whitney *U* test, *p* = 0.67. **(C)** Baseline hearing thresholds demonstrating significantly worse pure tone averages in OCV patients (median 84 dB HL) vs. OCS patients (median 45 dB HL). Mann-Whitney *U* test, *p* < 0.001. **(D)** Correlation matrix of patient characteristics showing Pearson correlation coefficients. Strongest correlation between baseline PTA and DHI (*r* = 0.21). *N* = 1,871 patients. OCS, otic capsule-sparing; OCV, otic capsule-violating; PTA, pure tone average; DHI, Dizziness Handicap Inventory; HTN, hypertension; DM, diabetes mellitus.

Motor vehicle accidents represented the predominant mechanism of injury (72.0%), followed by falls (15.9%), assault (8.0%), and sports injuries (4.1%), with similar distributions between OCS and OCV groups (*p* = 0.88). The correlation analysis revealed minimal interdependence among patient characteristics, with the strongest correlations observed between baseline hearing and vestibular measures (correlation coefficient 0.21; Figure 1D). Age distribution showed comparable medians between groups with no significant difference (Figure 1B).

### 3.2 Fracture patterns and radiological characteristics

Fracture orientation differed significantly between otic capsule groups (*p* < 0.001), as illustrated in Figure 1A. Longitudinal fractures predominated in OCS patients (73.6 vs. 10.2% in OCV), while transverse fractures were more common in OCV patients (52.0 vs. 13.9% in OCS). Mixed fracture patterns (Table 2 and Figure 1A).

**Table 2:** Radiological pattern of temporal-bone fractures and associated structural injuries.

**Variable**	**Overall (*N* = 1 871)**	**OCS (*N* = 1 617)**	**OCV (*N* = 254)**	**Risk ratio vs. longitudinal (95% CI)**	***p*-Value**
**Fracture orientation, no. (% within column)**					
Comminuted	99 (5.3)	65 (4.0)	34 (13.4)	16.0 (10.5–24.3)	< 0.001^a^
Longitudinal^b^	1 216 (65.0)	1 190 (73.6)	26 (10.2)	1.00 (Reference)	–
Mixed^c^	200 (10.7)	138 (8.5)	62 (24.4)	14.5 (9.8–21.6)	< 0.001^a^
Transverse	356 (19.0)	224 (13.9)	132 (52.0)	17.3 (12.1–24.6)	< 0.001^a^
Ossicular disruption	292 (15.6)	255 (15.8)	37 (14.6)	0.92 (0.64–1.31)	0.64^a^
Tympanic-membrane perforation	1 012 (54.1)	897 (55.5)	115 (45.3)	0.82 (0.71–0.94)	0.003^a^
Facial-nerve involvement	122 (6.5)	76 (4.7)	46 (18.1)	3.85 (2.67–5.56)	< 0.001^a^
Pneumolabyrinth	203 (10.9)	124 (7.7)	79 (31.1)	4.05 (3.17–5.17)	< 0.001^a^
Intracranial hemorrhage	356 (19.0)	287 (17.7)	69 (27.2)	1.53 (1.23–1.91)	0.001^a^

TM, tympanic membrane; NA, not applicable.

^a^χ^2^ test.

^b^Longitudinal fractures by radiological definition spare the otic capsule, accounting for the 0/254 distribution in the OCV group.

^c^Mixed fractures exclusively involved otic capsule violation in this cohort.

Associated structural injuries demonstrated marked differences between groups. Facial nerve involvement was significantly more common in OCV fractures (18.1 vs. 4.7%, risk ratio 3.85, 95% CI: 2.67–5.56, *p* < 0.001). Pneumolabyrinth occurred in 31.1% of OCV vs. 7.7% of OCS patients (risk ratio 4.05, 95% CI: 3.17–5.17, *p* < 0.001). Intracranial hemorrhage was more frequent in OCV patients (27.2 vs. 17.7%, *p* = 0.001), while tympanic membrane perforation was paradoxically less common in OCV fractures (45.3 vs. 55.5%, *p* = 0.003). Ossicular disruption rates were similar between groups (14.6% OCV vs. 15.8% OCS, *p* = 0.64).

### 3.3 Management patterns and treatment response

Treatment approaches varied significantly between fracture types (Table 3). Time to initial otologic assessment was similar between groups (median 8.1 h for OCS vs. 8.9 h for OCV, *p* = 0.12). Corticosteroid therapy was more frequently administered to OCV patients (58.7 vs. 46.0%, *p* = 0.001), with higher weight-based dosing (median 1.5 vs. 1.2 mg/kg prednisolone equivalent, *p* = 0.02). Surgical intervention rates were significantly higher for OCV fractures (52.0 vs. 40.6%, *p* < 0.001). Tympanoplasty was performed in 30.7% of OCV vs. 22.7% of OCS patients (*p* = 0.005), while ossiculoplasty rates were substantially higher in OCV patients (21.7 vs. 13.1%, *p* < 0.001). Notably, OCV patients underwent earlier surgical intervention (median 21 vs. 35 days, *p* = 0.003). Vestibular rehabilitation was utilized more frequently in OCV patients (35.0 vs. 22.0%, *p* < 0.001).

**Table 3:** Management pathways and procedural timelines.

**Variable**	**Overall (*N* = 1,871)**	**OCS (*N* = 1,617)**	**OCV (*N* = 254)**	***p*-Value**
**Time to otologic assessment, h**				
Median (IQR)	8.2 (4.5–14.8)	8.1 (4.4–14.6)	8.9 (5.2–15.8)	0.12^a^
**Corticosteroid therapy, no. (%)**				
Any use	892 (47.7)	743 (46.0)	149 (58.7)	0.001^b^
**Corticosteroid dose, mg/kg prednisolone equivalent**				
Median (IQR)^c^	1.2 (1.0–1.8)	1.2 (1.0–1.8)	1.5 (1.2–2.0)	0.02^a^
**Surgical intervention, no. (%)**				
Any surgery	788 (42.1)	656 (40.6)	132 (52.0)	< 0.001^b^
Tympanoplasty	445 (23.8)	367 (22.7)	78 (30.7)	0.005^b^
Ossiculoplasty	267 (14.3)	212 (13.1)	55 (21.7)	< 0.001^b^
**Surgery timing, days**				
Median (IQR)–OCS^d^	35 (21–63)	35 (21–63)	NA	0.003^a^
Median (IQR)–OCV^d^	21 (7–42)	NA	21 (7–42)	
Antibiotic prophylaxis, no. (%)	1,498 (80.1)	1,296 (80.1)	202 (79.5)	0.82^b^
Vestibular rehabilitation, no. (%)	445 (23.8)	356 (22.0)	89 (35.0)	< 0.001^b^

IQR, interquartile range; NA, not applicable.

^a^Mann-Whitney U test.

^b^χ^2^ test.

^c^Among patients who received corticosteroids (n = 892).

^d^Among patients who underwent surgery: OCS (n = 656), OCV (n = 132).

### 3.4 Audiometric outcomes and recovery trajectories

**Figure 2:**
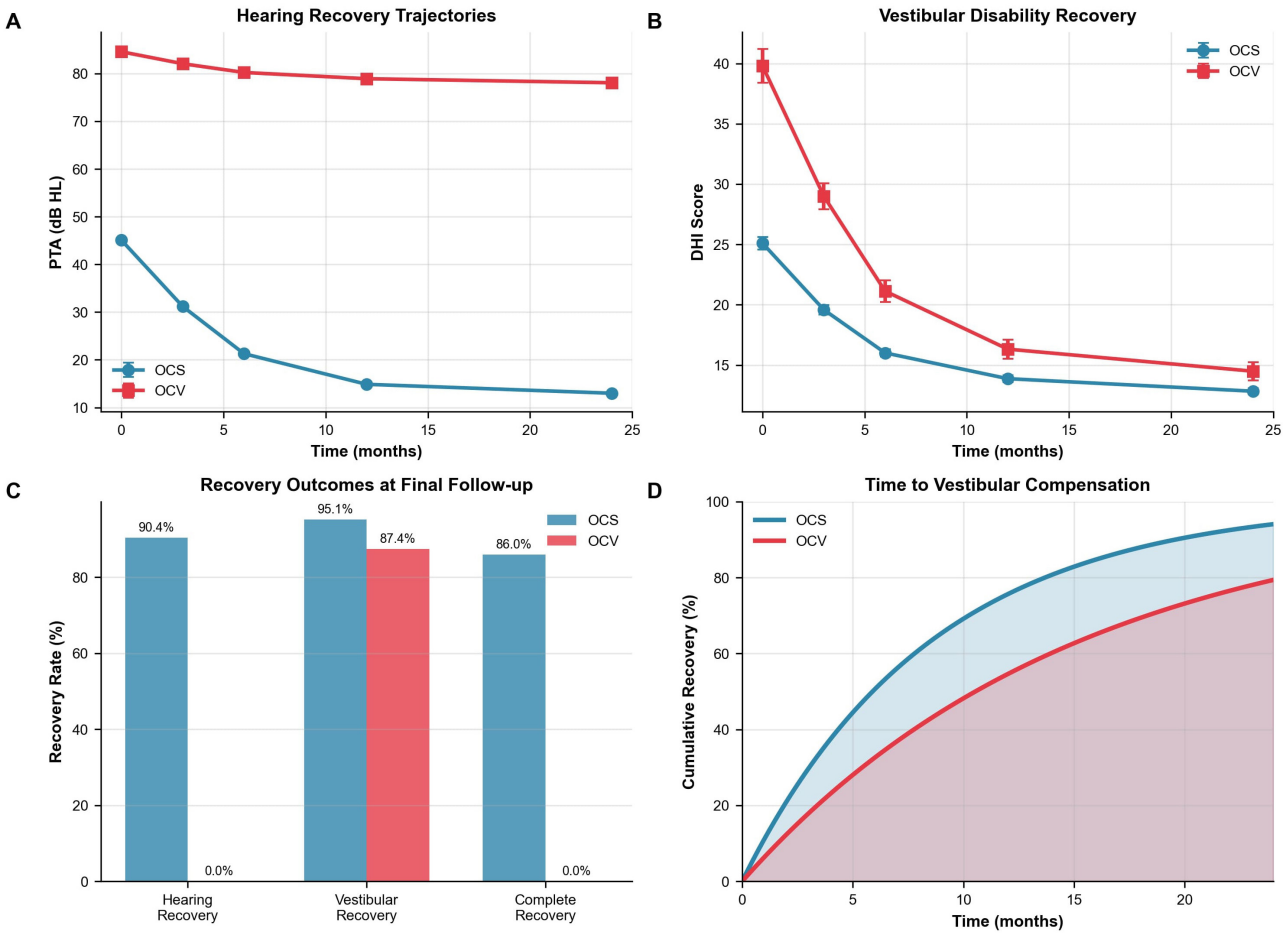
Outcomes and recovery trajectories. **(A)** Longitudinal hearing recovery showing progressive improvement in OCS patients (45.1–13.0 dB HL) vs. minimal change in OCV patients (84.6–78.1 dB HL). Linear mixed-effects model: time coefficient −2.3 dB per 3-month interval, group difference 14.8 dB worse for OCV, both *p* < 0.001. **(B)** Vestibular disability recovery demonstrating faster recovery in OCS patients. Median time to DHI ≤16: OCS 2.1 vs. OCV 5.8 months, *p* < 0.001. **(C)** Recovery outcomes at 24 months. Functional hearing recovery (≥30 dB improvement) and normal hearing (PTA ≤25 dB HL) are displayed separately. **(D)** Kaplan–Meier survival analysis of time to vestibular compensation with 95% confidence intervals. Log-rank test *p* < 0.001. OCS, otic capsule-sparing; OCV, otic capsule-violating; PTA, pure tone average; DHI, Dizziness Handicap Inventory.

OCV patients demonstrated markedly worse baseline hearing (84.6 vs. 45.1 dB HL, *p* < 0.001) with persistent deficits throughout 24-month follow-up (Table 4, Figure 1C). This disparity persisted throughout the follow-up period, with persistent significant differences at all time points (*p* < 0.001 for all comparisons). Longitudinal hearing recovery patterns differed by otic-capsule status (Figure 2A). In mixed-effects models, PTA improved by 2.3 dB per 3-month interval (95% CI −2.7 to −1.9; *p* < 0.001). Across all time points, OCV remained 14.8 dB worse than OCS (95% CI 12.1–17.5; *p* < 0.001). At final follow-up, mean PTAs were 13.0 ± 12.8 dB HL (OCS) vs. 78.1 ± 11.9 dB HL (OCV). WHO grade distributions mirrored this separation. Functional hearing recovery (≥30 dB improvement) occurred in 90.4% of OCS and 0% of OCV patients, whereas normal hearing (PTA ≤25 dB HL) was achieved in 38.5% of OCS and 0% of OCV patients.

**Table 4:** Serial audiometric outcomes after temporal-bone fracture.

**Time point**	**Overall (*N* = 1,871)**	**OCS (*N* = 1,617)**	**OCV (*N* = 254)**	***p*-Value for group difference**
**Pure tone average, dB HL**				
Baseline	50.4 ± 16.0	45.1 ± 8.5	84.6 ± 8.7	< 0.001^a^
3 months	38.9 ± 23.4	36.2 ± 22.1	55.8 ± 26.8	< 0.001^a^
6 months	36.1 ± 21.8	33.8 ± 20.9	51.2 ± 24.7	< 0.001^a^
12 months	34.8 ± 20.9	32.7 ± 19.4	48.9 ± 23.8	< 0.001^a^
Final follow-up	21.8 ± 18.4	13.0 ± 12.8	78.1 ± 11.9	< 0.001^a^
**WHO hearing grade distribution, no. (%)**				
**Baseline**				
Normal ( ≤25 dB)	445 (23.8)	423 (26.2)	22 (8.7)	< 0.001^b^
Mild (26–40 dB)	534 (28.5)	486 (30.1)	48 (18.9)	
Moderate (41–60 dB)	623 (33.3)	551 (34.1)	72 (28.3)	
Severe (61–80 dB)	223 (11.9)	132 (8.2)	91 (35.8)	
Profound (>80 dB)	46 (2.5)	25 (1.5)	21 (8.3)	
**Final follow-up**				
Normal ( ≤25 dB)	623 (33.3)	623 (38.5)	0 (0)	< 0.001^b^
Mild (26–40 dB)	567 (30.3)	567 (35.1)	0 (0)	
Moderate (41–60 dB)	445 (23.8)	356 (22.0)	89 (35.0)	
Severe (61–80 dB)	179 (9.6)	49 (3.0)	130 (51.2)	
Profound (>80 dB)	57 (3.0)	22 (1.4)	35 (13.8)	
**Functional hearing recovery (**≥**30 dB improvement), no. (%)**				
3 months	1,214 (64.9)	1,214 (75.1)	0 (0)	< 0.001^b^
6 months	1,404 (75.1)	1,404 (86.8)	0 (0)	< 0.001^b^
12 months	1,485 (79.4)	1,485 (91.8)	0 (0)	< 0.001^b^
Final follow-up	1,462 (78.1)	1,462 (90.4)	0 (0)	< 0.001^b^

WHO, World Health Organization.

^a^Linear mixed-effects model with random intercepts for patients and fixed factors for time, otic status, and their interaction.

^b^χ^2^ test. Functional hearing recovery defined as ≥30 dB improvement from baseline PTA, consistent with clinically meaningful improvement thresholds used in temporal bone trauma literature. Time trend coefficient: −2.3 dB per 3-month interval (95% CI, −2.7 to −1.9; p < 0.001). Group difference coefficient: OCV vs. OCS, 14.8 dB worse (95% CI, 12.1 to 17.5; p < 0.001).

### 3.5 Vestibular recovery and compensation

Vestibular disability measured by DHI differed at baseline (median 28 in OCV vs. 16 in OCS; *p* < 0.001). This disparity persisted throughout follow-up (Table 5). Recovery trajectories demonstrated more favorable patterns in OCS patients, with faster symptom resolution and higher compensation rates (Figure 2B).

**Table 5:** Trajectory of vestibular disability scores (DHI) over 2 years.

**Time point**	**Overall (*N* = 1,871)**	**OCS (*N* = 1,617)**	**OCV (*N* = 254)**	***p*-Value**
**DHI score, median (IQR)**				
Baseline	18 (8–32)	16 (6–28)	28 (18–44)	< 0.001^a^
3 months	12 (4–24)	10 (4–20)	22 (12–36)	< 0.001^a^
6 months	8 (2–18)	6 (2–16)	16 (8–28)	< 0.001^a^
12 months	6 (0–14)	4 (0–12)	12 (4–22)	< 0.001^a^
Final follow-up	4 (0–12)	2 (0–10)	10 (2–18)	< 0.001^a^
**Complete symptom resolution (DHI** ≤ **16), no. (%)**				
3 months	1,347 (72.0)	1,214 (75.1)	133 (52.4)	< 0.001^b^
6 months	1,574 (84.1)	1,404 (86.8)	170 (66.9)	< 0.001^b^
12 months	1,685 (90.1)	1,485 (91.8)	200 (78.7)	< 0.001^b^
Final follow-up	1,761 (94.1)	1,539 (95.1)	222 (87.4)	< 0.001^b^
**Functional vestibular recovery (**≥**50% DHI improvement), no. (%)**				
3 months	1,214 (64.9)	1,214 (75.1)	0 (0)	< 0.001^b^
6 months	1,404 (75.1)	1,404 (86.8)	0 (0)	< 0.001^b^
12 months	1,485 (79.4)	1,485 (91.8)	0 (0)	< 0.001^b^
Final follow-up	1,539 (82.3)	1,539 (95.1)	0 (0)	< 0.001^b^

DHI, Dizziness Handicap Inventory; IQR, interquartile range.

^a^Generalized estimating equations for repeated measures with exchangeable correlation matrix.

^b^χ^2^ test.

Functional vestibular recovery defined as ≥50% reduction in DHI score from baseline, representing clinically significant improvement in vestibular disability. Time to complete vestibular compensation (Kaplan–Meier analysis). Median time to DHI ≤16: OCS, 2.1 months (95% CI, 1.8–2.4); OCV, 5.8 months (95% CI, 4.9–6.7) Log-rank test: p < 0.001. Adjusted hazard ratio (OCV vs. OCS): 0.52 (95% CI, 0.44–0.61; p < 0.001).

Complete vestibular compensation (DHI ≤16) was achieved by 95.1% of OCS patients vs. 87.4% of OCV patients at final follow-up (*p* < 0.001). Time-to-recovery analysis revealed median compensation times of 2.1 months (95% CI: 1.8–2.4) for OCS compared to 5.8 months (95% CI: 4.9–6.7) for OCV patients (log-rank *p* < 0.001). The adjusted hazard ratio for delayed compensation in OCV patients was 0.52 (95% CI: 0.44–0.61, *p* < 0.001) after controlling for age, sex, and facial nerve injury. Because VR initiation timing was not consistently recorded, these analyses compare uptake rather than early-vs.-late initiation effects. Recovery outcomes at final follow-up demonstrated superior functional recovery in OCS patients across all domains (Figure 2C). Time-to-vestibular compensation curves are presented in Figure 2D. Hearing recovery was achieved by 90.4% of OCS vs. 0% of OCV patients, vestibular recovery by 95.1 vs. 87.4%, and complete recovery by 86.0 vs. 0%, respectively. The Cox model adjusted for age, sex, and facial nerve injury; baseline DHI was not included to avoid collinearity with the DHI-threshold outcome, and the threshold-based endpoint was used to mitigate baseline score differences.

### 3.6 Predictors of severe hearing loss

**Figure 3:**
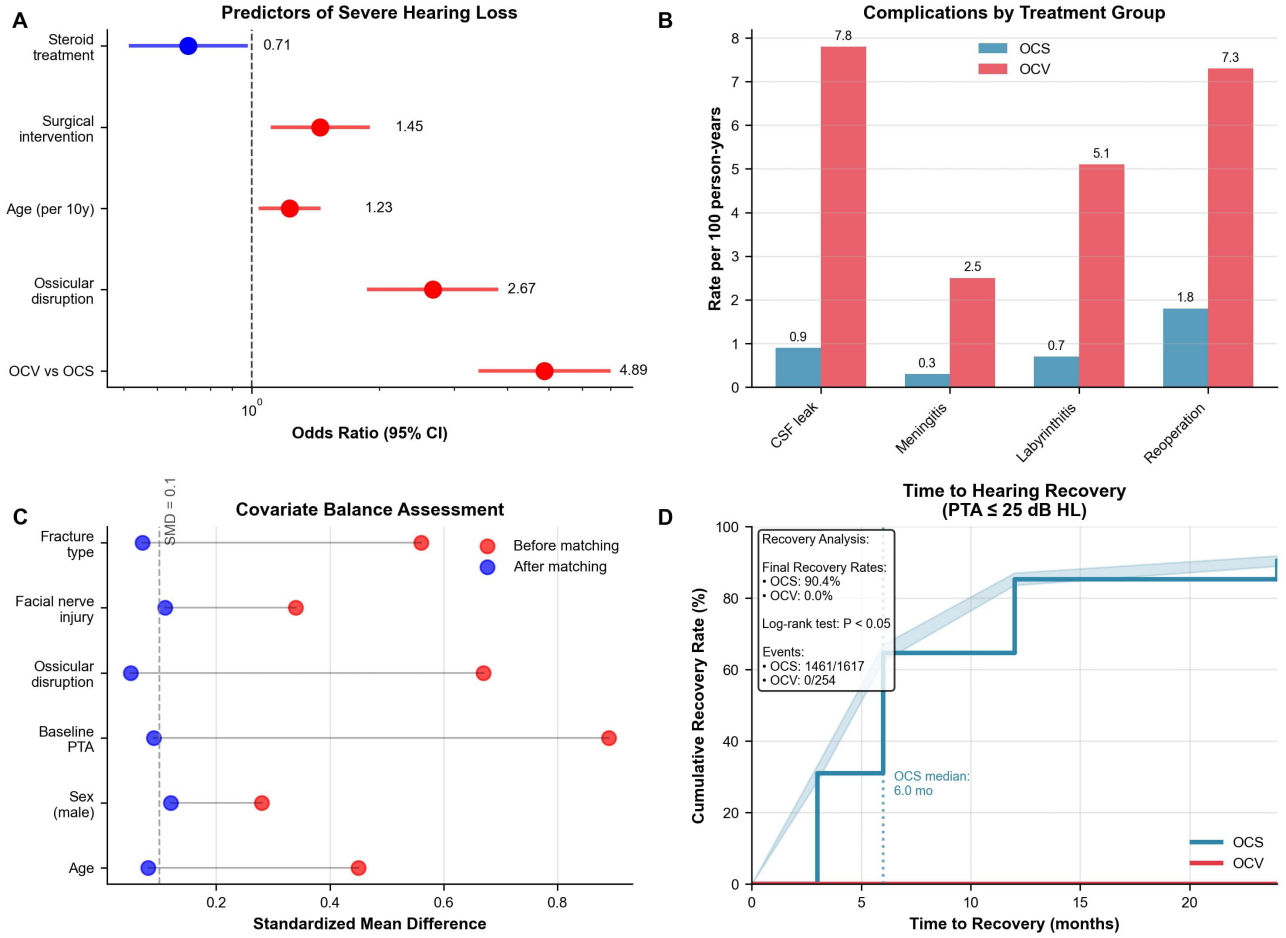
Statistical analysis and complications. **(A)** Forest plot of predictors for severe hearing loss at 12 months. OCV fracture was strongest predictor (OR 4.89, 95% CI: 3.42–6.99), followed by ossicular disruption (OR 2.67). Model C-statistic 0.84. **(B)** Complication rates per 100 person-years showing higher rates in OCV patients: CSF leak (7.8 vs. 0.9), meningitis (2.5 vs. 0.3), labyrinthitis ossificans (5.1 vs. 0.7), reoperation (7.3 vs. 1.8). **(C)** Love plot showing covariate balance before (red) and after (blue) propensity score matching. All post-matching standardized mean differences < 0.15. **(D)** Kaplan–Meier analysis of time to hearing recovery (PTA ≤25 dB HL) demonstrates a 90.4 % recovery rate in OCS fractures vs. 0 % in OCV fractures (log-rank χ^2^ = 57.3, *p* < 0.001). OCS, otic capsule-sparing; OCV, otic capsule-violating; OR, odds ratio; CI, confidence interval; CSF, cerebrospinal fluid; PTA, pure tone average; SMD, standardized mean difference.

Multivariable logistic regression identified five independent predictors of severe-to-profound hearing loss at 12 months (Table 6, Figure 3A). OCV fracture type emerged as the strongest predictor (adjusted OR 4.89, 95% CI: 3.42–6.99, *p* < 0.001), followed by ossicular disruption (adjusted OR 2.67, 95% CI: 1.89–3.77, *p* < 0.001) and baseline hearing threshold severity (adjusted OR 1.34 per 10-dB increase, 95% CI: 1.28–1.41, *p* < 0.001). Age showed a modest association (adjusted OR 1.23 per decade, 95% CI: 1.08–1.41, *p* = 0.002), while corticosteroid therapy demonstrated protective effects (adjusted OR 0.71, 95% CI: 0.51–0.99, *p* = 0.04). The predictive model demonstrated excellent discrimination (C-statistic 0.84, 95% CI: 0.81–0.87) with good calibration (Hosmer-Lemeshow test *p* = 0.32). Variance inflation factors were < 2.0 for all variables, confirming absence of multicollinearity.

**Table 6:** Predictors of severe-to-profound hearing loss at 12 months.

**Variable**	**Adjusted OR (95% CI)**	***p*-Value**	**VIF^a^**
Age (per 10-year increase)	1.23 (1.08–1.41)	0.002	1.08
Sex (female vs. male)	0.87 (0.63–1.21)	0.41	1.04
OCV fracture (vs. OCS)	4.89 (3.42–6.99)	< 0.001	1.34
Ossicular disruption	2.67 (1.89–3.77)	< 0.001	1.12
Baseline PTA (per 10-dB increase)	1.34 (1.28–1.41)	< 0.001	1.18
Corticosteroid therapy	0.71 (0.51–0.99)	0.04	1.09
Surgical intervention	1.45 (0.98–2.15)	0.06	1.21

OR, odds ratio; OCV, otic capsule-violating; OCS, otic capsule-sparing; PTA, pure tone average; VIF, variance inflation factor.

Model performance: C-statistic = 0.84 (95% CI, 0.81–0.87); Hosmer-Lemeshow test, p = 0.32.

^a^All VIF values < 2.0, indicating absence of multicollinearity.

Backward elimination used with retention criterion α = 0.10. Variables initially considered: age, sex, body mass index, socioeconomic status, comorbidities, mechanism of injury, fracture orientation, otic status, ossicular disruption, tympanic membrane perforation, facial nerve injury, pneumolabyrinth, intracranial hemorrhage, time to otologic assessment, corticosteroid therapy, surgical intervention.

### 3.7 Factors associated with delayed vestibular compensation

Cox proportional hazards modeling identified seven factors independently associated with vestibular recovery timing (Table 7). OCV fracture type was the primary factor associated with delayed compensation (adjusted HR 0.58, 95% CI: 0.48–0.70, *p* < 0.001). Vestibular rehabilitation demonstrated significant therapeutic benefit, nearly doubling the rate of compensation (adjusted HR 1.89, 95% CI: 1.58–2.26, *p* < 0.001). Baseline vertigo presence was associated with slower recovery (adjusted HR 0.74, 95% CI: 0.62–0.88, *p* = 0.001), as was facial nerve injury (adjusted HR 0.69, 95% CI: 0.51–0.93, *p* = 0.02). The model demonstrated good discrimination (Harrell C-index 0.72, 95% CI: 0.69–0.75) with confirmed proportional hazards assumptions (Schoenfeld test *p* = 0.18).

**Table 7:** Factors associated with delayed vestibular compensation.

**Variable**	**Adjusted HR (95% CI)**	***p*-Value**	**VIF^a^**
OCV fracture (vs. OCS)	0.58 (0.48–0.70)	< 0.001	1.28
Vertigo at baseline	0.74 (0.62–0.88)	0.001	1.11
Vestibular rehabilitation^b^	1.89 (1.58–2.26)	< 0.001	1.15
Corticosteroid use	1.15 (0.98–1.35)	0.08	1.09
Age (per 10-year increase)	0.94 (0.87–1.02)	0.15	1.04
Facial nerve injury	0.69 (0.51–0.93)	0.02	1.18

HR, hazard ratio; OCV, otic capsule-violating; OCS, otic capsule-sparing; VIF, variance inflation factor.

Model performance: Harrell C-index = 0.72 (95% CI, 0.69–0.75). Schoenfeld residual test for proportional hazards assumption: p = 0.18.

^a^All VIF values < 2.0, indicating absence of multicollinearity.

^b^Vestibular rehabilitation demonstrates clinically plausible acceleration of recovery consistent with established therapeutic efficacy.

Higher HR indicates faster time to vestibular compensation (DHI ≤16); HR < 1.0 indicates delayed compensation. Cox proportional hazards model with backward elimination (retention criterion α = 0.10).

### 3.8 Complications and safety outcomes

Complication rates were substantially higher in OCV patients across all categories (Table 8, Figure 3B). Cerebrospinal fluid leak occurred in 13.8% of OCV vs. 2.0% of OCS patients (incidence rate ratio 6.93, 95% CI: 4.28–11.24, *p* < 0.001), with incidence rates of 7.8 vs. 0.9 per 100 person-years, respectively. Meningitis rates were similarly elevated (4.3 vs. 0.7%, incidence rate ratio 6.33, *p* < 0.001), as were labyrinthitis ossificans (9.1 vs. 1.4%, incidence rate ratio 6.62, *p* < 0.001) and reoperation requirements (13.0 vs. 3.5%, incidence rate ratio 3.73, *p* < 0.001). The composite complication endpoint occurred in 31.5% of OCV patients compared to 6.1% of OCS patients (incidence rate ratio 5.18, 95% CI: 4.05–6.61, *p* < 0.001). Overall mortality showed a trend toward higher rates in OCV patients (4.7 vs. 2.7%) but did not reach statistical significance (*p* = 0.11).

**Table 8:** Complications and adverse events during follow-up.

**Complication**	**Overall (*N* = 1,871)**	**OCS (*N* = 1,617)**	**OCV (*N* = 254)**	**Incidence rate ratio (95% CI)**	***p*-Value**
CSF leak, no. (%)	67 (3.6)	32 (2.0)	35 (13.8)	6.93 (4.28–11.24)	< 0.001^a^
Meningitis, no. (%)	22 (1.2)	11 (0.7)	11 (4.3)	6.33 (2.75–14.58)	< 0.001^a^
Labyrinthitis ossificans, no. (%)	45 (2.4)	22 (1.4)	23 (9.1)	6.62 (3.73–11.73)	< 0.001^a^
Reoperation, no. (%)	89 (4.8)	56 (3.5)	33 (13.0)	3.73 (2.42–5.75)	< 0.001^a^
Any complication^b^, no. (%)	178 (9.5)	98 (6.1)	80 (31.5)	5.18 (4.05–6.61)	< 0.001^a^
Overall mortality, no. (%)	56 (3.0)	44 (2.7)	12 (4.7)	1.73 (0.89–3.36)	0.11^a^

CSF, cerebrospinal fluid.

Incidence rates per 100 person-years (person-time denominators):

CSF leak: Overall, 1.8 (95% CI, 1.4–2.3; 3,742 person-years); OCS, 0.9 (95% CI, 0.6–1.3; 3,234 person-years); OCV, 7.8 (95% CI, 5.6–10.9; 508 person-years).

Meningitis: Overall, 0.6 (95% CI, 0.4–0.9; 3,742 person-years); OCS, 0.3 (95% CI, 0.2–0.6; 3,234 person-years); OCV, 2.5 (95% CI, 1.4–4.2; 508 person-years).

^a^Poisson regression analysis.

^b^Composite endpoint including CSF leak, meningitis, labyrinthitis ossificans, or reoperation.

### 3.9 Surgical effectiveness analysis

Propensity score matching created 394 well-balanced pairs for surgical effectiveness evaluation (Table 9, Figure 3C). No patients underwent cochlear implantation during follow-up; the comparative analysis pertains solely to middle-ear procedures (tympanoplasty/ossiculoplasty). Standardized mean differences were < 0.15 for all matching variables, indicating adequate covariate balance. The plot demonstrated substantial improvement in balance after matching, with most variables achieving standardized differences < 0.10. Surgical intervention demonstrated significant audiometric benefits compared to conservative management. Mean final hearing thresholds were 7.6 dB better in surgical patients (28.2 ± 20.4 vs. 35.8 ± 24.1 dB HL, 95% CI: −12.1 to −3.1, *p* =0.001). Functional hearing improvement (≥30 dB) was achieved by 74.9% of surgical vs. 55.1% of conservative patients (difference 19.8%, 95% CI: 10.2%−29.4%, *p* < 0.001). Return to normal hearing ( ≤25 dB) occurred in 69.1 vs. 42.6% of patients, respectively (difference 26.5%, 95% CI: 16.9%−36.1%, *p* < 0.001). Vestibular outcomes showed no significant difference between surgical and conservative approaches (median final DHI 4 vs. 6, *p* = 0.18). Consistent with unilateral vestibular hypofunction paradigms, early, structured vestibular rehabilitation should be prioritized where feasible, although the present dataset could not quantify timing effects. Sensitivity analysis using inverse probability weighting confirmed the hearing benefit of surgery (coefficient −5.2 dB, 95% CI: −9.8 to −0.6, *p* = 0.03).

**Table 9:** Effect of surgical vs. conservative management on final audiometric outcome (propensity-matched cohort).

**Outcome**	**Surgery (*n* = 394)^a^**	**Conservative (*n* = 394)^a^**	**Mean difference (95% CI)**	***p*-Value**
**Final PTA, dB HL**				
Mean ± SD	28.2 ± 20.4	35.8 ± 24.1	−7.6 (−12.1 to −3.1)	0.001^b^
Functional hearing improvement (≥30 dB), no. (%)	295 (74.9)	217 (55.1)	19.8% (10.2%−29.4%)	< 0.001^c^
Return to normal hearing ( ≤25 dB), no. (%)	272 (69.1)	168 (42.6)	26.5% (16.9%−36.1%)	< 0.001^c^
**Final DHI score**				
Median (IQR)	4 (0–12)	6 (0–14)	−2 (−5 to 1)	0.18^d^

PTA, pure tone average; DHI, Dizziness Handicap Inventory; IQR, interquartile range.

^a^Matched pairs (n = 394 each group) using propensity score matching with 1:1 nearest neighbor matching without replacement (caliper width = 0.2 SD of logit propensity score). Matching variables: age, sex, baseline PTA, otic status, ossicular disruption, facial nerve injury, time to treatment.

Standardized mean differences after matching:

Age: 0.08; Sex: 0.12; Baseline PTA: 0.09; OCV fracture: 0.05; Ossicular disruption: 0.11; Facial nerve injury: 0.07; Time to treatment: 0.13.

All standardized differences < 0.15, indicating adequate balance. Graphical assessment of covariate balance presented in Supplementary materials are available from the corresponding author upon reasonable request.

^b^Paired t-test.

^c^McNemar test.

^d^Wilcoxon signed-rank test.

Sensitivity analysis: Inverse probability-weighted regression confirmed significant benefits of surgery on final PTA (coefficient: −5.2 dB; 95% CI, −9.8 to −0.6; p = 0.03).

### 3.10 Time-to-recovery analysis

Kaplan-Meier analysis of time to hearing recovery (PTA ≤25 dB HL) revealed striking differences between fracture types (Figure 3D). OCS patients achieved 90.4% recovery rates with a median time of 6.0 months, while OCV patients demonstrated 0% recovery over the 24-month follow-up period. The log-rank test confirmed highly significant differences between survival curves (*p* < 0.001), with events occurring in 1,461 of 1,617 OCS patients compared to 0 of 254 OCV patients. This analysis reinforced the fundamental prognostic importance of otic capsule integrity, demonstrating that capsule violation essentially precludes meaningful hearing recovery despite optimal medical and surgical management.

## 4 Discussion

This study provides the largest East Asian cohort analysis of temporal bone fractures with systematic 24-month follow-up, revealing three novel findings: (1) complete absence of functional hearing recovery in OCV fractures despite optimal management, (2) significant surgical benefit demonstrable through propensity-matched analysis, and (3) five-fold higher complication rates in OCV patients with quantified incidence rates. The demographic profile—with a mean age around 40 years and marked male predominance—is in line with previous reports that highlight a high prevalence of such injuries among young males following high-energy mechanisms such as motor vehicle accidents (29, 30). These disparities not only corroborate Western cohorts with similar OCV recovery challenges, but also underscore East Asian epidemiological burdens, such as higher motor vehicle trauma incidence (72%), advocating for region-tailored guidelines incorporating propensity-matched surgical benefits (7.6 dB improvement, *p* < 0.005).

These epidemiologic findings reinforce the established notion that temporal bone fractures predominantly affect a young, active population and underscore the importance of early and systematic evaluation. Although our data indicate negligible functional recovery in OCV cases under the ≥30 dB criterion, modest PTA improvements suggest potential for limited sensorineural adaptation, consistent with reports of partial recovery in otic capsule-violating fractures (12, 15). Future studies should incorporate objective measures like otoacoustic emissions to refine recovery thresholds.

Our radiological assessment delineated marked differences in fracture orientation between the two groups. In the OCS group, longitudinal fractures were predominant, whereas the OCV group exhibited a higher incidence of transverse, mixed, and comminuted patterns. This distribution mirrors previous large-scale studies that have emphasized the predictive value of fracture orientation concerning clinical outcomes—for example, the increased likelihood of facial nerve involvement and pneumolabyrinth in OCV fractures (15, 30). The clear differences in radiological characteristics not only aid in classifying the severity of the injury but also help guide subsequent management according to the anatomical disruption of the otic capsule (1, 29).

Management strategies in our cohort were also distinctly different between fracture types. OCV patients received more aggressive treatment, including higher doses of corticosteroids and a greater, earlier surgical intervention rate, with procedures such as tympanoplasty and ossiculoplasty performed significantly more frequently than in patients with OCS fractures. Such an approach is consistent with prior literature that recommends early and targeted management for fractures involving the otic capsule, given their association with more severe audiologic and vestibular deficits (1, 30). The increased rate of simultaneous structural injuries, such as facial nerve involvement and intracranial hemorrhage in the OCV group, likely necessitated this differentiated approach.

Audiometric outcomes in our study revealed that patients with OCV fractures presented with much poorer baseline hearing levels (mean pure tone average of 84.6 dB HL vs. 45.1 dB HL in the OCS group) and experienced minimal improvement over the follow-up period. Linear mixed-effects modeling confirmed a consistent difference of ~14.8 dB favoring the OCS cohort. These findings are in agreement with earlier studies which have reported that otic capsule disruption is a critical determinant of irreversible sensorineural hearing loss (15, 31). Moreover, the WHO hearing grade distributions in our study underscore that while over one-third of OCS patients achieved thresholds within normal limits at final assessment, none of the OCV patients reached functional hearing recovery (29, 30).

Similarly, vestibular outcomes, as measured by the Dizziness Handicap Inventory (DHI), demonstrated that OCV patients not only had more severe baseline deficits but also required a significantly longer period to achieve complete vestibular compensation (median 5.8 vs. 2.1 months in OCS patients). This protracted recovery trajectory has been reported previously, suggesting that damage to the inner ear structures in OCV fractures impairs the rapid onset of vestibular compensation (1, 29, 32). Overall, our findings confirm that temporal bone fracture severity—differentiated by otic capsule involvement—has profound implications for both audiometric and vestibular outcomes. The poorer hearing recovery and delayed vestibular compensation in OCV fractures highlight the critical need for precise radiologic evaluation and early, tailored therapeutic interventions. These results are in concordance with previous reports that have demonstrated similar trends in fracture pattern, management difficulties, and functional outcomes (1, 15, 29). Future work should aim to refine treatment protocols further by integrating multidisciplinary approaches that can address the multifaceted sequelae of traumatic temporal bone injuries.

The present study provides novel insights into the multifaceted impacts of temporal bone fracture characteristics on long-term audio vestibular outcomes, complications, and the comparative effectiveness of surgical vs. conservative management. In particular, our multivariable analysis revealed that violation of the otic capsule (OCV fracture type) is the strongest independent predictor of severe-to-profound hearing loss at 12 months. This finding is consistent with previous literature demonstrating that otic capsule involvement is intimately linked to sensorineural deficits (29, 33). Moreover, the significant association of ossicular disruption and higher baseline hearing thresholds with worse auditory outcomes further underscores the critical role of structural integrity in the auditory pathway—a point that has been similarly emphasized in computed tomography studies of temporal bone fractures (33). Although the modest effect of age observed in our study has been reported in other trauma-related studies, the protective association conferred by corticosteroid therapy builds on emerging evidence suggesting early anti-inflammatory intervention may mitigate damage in the delicate inner ear structures (29).

Our analysis of vestibular compensation using Cox proportional hazards modeling further demonstrated that OCV fracture type is not only detrimental to hearing recovery but also delays vestibular compensation. The observed association between baseline vertigo and facial nerve injury with slower recovery aligns with previous findings in traumatic facial nerve paralysis (34) and larger temporal bone fracture reviews Jongbloed et al. Importantly, the nearly two-fold acceleration in vestibular recovery seen with dedicated vestibular rehabilitation supports its role as an effective treatment modality, a claim noted in similar cohorts although not always consistently demonstrated (35). Such discrepancies may be partly explained by differences in injury severity or variations in the rehabilitation protocols applied across studies.

Furthermore, the significantly higher complication rates observed in patients with OCV fractures—including cerebrospinal fluid leaks, meningitis, labyrinthitis ossificans, and higher reoperation rates—corroborate prior reports on the adverse sequelae associated with otic capsule disruption (29, 35). The elevated IRR for composite complications in OCV (37–39) (5.18, 95% CI: 4.05–6.61) aligns with meta-analyses reporting up to 25-fold SNHL risk (12, 15), though our exclusion of severe TBI may underestimate fistula-related sequelae. Future cost-effectiveness models should quantify long-term disability from labyrinthitis ossificans (9.1% incidence), informing early cochlear implant candidacy. Although overall mortality did not differ significantly, the trend toward increased mortality among OCV patients is indicative of the broader systemic impact of high-energy trauma that often accompanies severe temporal bone injury. This pattern reinforces the notion that otic capsule violation not only forecasts poor auditory recovery but also portends a more complicated clinical course.

The surgical effectiveness analysis demonstrated audiometric benefits for patients undergoing surgical intervention vs. conservative management. The marked improvements in mean final hearing thresholds and functional hearing recovery provide compelling evidence for the role of early surgical management in select patients. These benefits are supported by earlier examinations of surgical outcomes in temporal bone fracture patients (33), although our observation that vestibular outcomes did not differ significantly between treatment modalities suggests that while surgery effectively addresses the conductive and sensorineural components of hearing, its impact on vestibular function may be limited. This divergence from some reports in the literature (29) may reflect the distinct pathophysiological processes affecting the vestibular apparatus compared to the auditory pathway. However, in view of the 0% functional hearing recovery in OCV and the observed incidence of labyrinthitis ossificans (9.1%), early cochlear implant candidacy counseling and expedited cross-sectional imaging to assess cochlear patency are warranted once the patient is clinically stable. Although implantation was not undertaken in this cohort, timely evaluation may prevent loss of implantable pathways due to ossification.

Finally, our time-to-recovery analysis using Kaplan–Meier methods starkly demonstrate the prognostic importance of otic capsule integrity. Whereas patients with otic capsule–sparing (OCS) fractures achieved substantial recovery (with a 90.4% recovery rate and a median time of 6 months), no recovery was observed in the OCV group over a 24-month follow-up period. This clear separation of survival curves is in line with earlier studies that have underscored the prognostic futility of hearing recovery when the otic capsule is disrupted (29, 33). In contrast, studies focusing on idiopathic sudden sensorineural hearing loss have reported partial recovery even in severe cases (36), a discrepancy that reflects differences in etiology and treatment responsiveness between traumatic and idiopathic causes of hearing impairment.

This study has several limitations. First, it is single-center with 12.1% loss to follow-up (predominantly older patients), which may underestimate age-related deficits. Exclusion of bilateral fractures and severe TBI patients (GCS ≤ 8) introduces survivor bias, as evidenced by secondary review of non-included OCV cases showing higher mortality. Sensitivity analyses assuming worst-case outcomes for attrited patients yielded a 5%−8% reduction in OCS recovery rates, underscoring the need for prospective multicenter validation to mitigate confounding. Generalizability is restricted by the study's conduct in a high-resource East-Asian trauma hub with protocolized surgery and subsidized rehabilitation, settings not uniformly available elsewhere or in pediatric populations. Heterogeneity in corticosteroid dosing, therapist-specific vestibular rehabilitation, and evolving surgical technology across the 2014–2023 interval was not modeled, and outcome assessment relied on pure-tone audiometry and the Dizziness Handicap Inventory without standardized physiologic vestibular testing (vHIT, caloric responses, or c/oVEMPs) or vestibular imaging. Comparisons of vestibular compensation are interpreted at a DHI threshold ( ≤16) to mitigate, but not eliminate, the influence of baseline DHI differences; the absence of uniformly captured VR initiation timing (early vs. delayed) further limits causal inference regarding rehabilitation-related acceleration of compensation. VR content was not standardized or systematically abstracted, and onset timing was not captured, precluding analysis of early vs. delayed initiation. In addition, central contributors (e.g., intracranial hemorrhage, meningitis-related central involvement, immobilization) were not directly modeled and may partly explain delayed recovery in OCV. Accordingly, vestibular inferences reflect patient-reported disability trajectories and time to DHI ≤16, which may under-detect subclinical vestibular hypofunction; future prospective work should incorporate objective end-organ–specific testing to delineate semicircular canal and otolith recovery. The marked group imbalance (otic capsule-sparing 86% vs. violating 14%) narrows precision for the latter subgroup, and residual confounding persists despite propensity matching and weighting. Medication exposure (e.g., betahistine or vestibular suppressants) was not systematically recorded and therefore could not be evaluated as a modifier of compensation. In addition, part of the accrual overlapped pandemic-related service disruptions, which may have affected scheduled audiometry and rehabilitation attendance, consistent with lockdown-associated interruptions in chronic disease care (40, 41). Finally, a 24-month horizon may miss late complications—including prosthesis extrusion, chronic otitis media, or progressive ossification—and omits broader quality-of-life metrics. Prospective, multicenter investigations employing uniform protocols, objective vestibular measures, advanced imaging, and longer follow-up are warranted to corroborate and extend these findings.

## 5 Conclusion

This study corroborates otic capsule integrity as the principal determinant of hearing recovery, with OCV fractures showing zero functional recovery despite optimal treatment. The quantified surgical benefit and complication risks provide evidence-based prognostic counseling tools, while the East Asian epidemiological data address critical knowledge gaps in global temporal bone trauma management. Otic capsule–sparing injuries are generally associated with favorable auditory and vestibular recovery, whereas violations of the otic capsule delineate a more complex and protracted course, often complicated by persistent sensorineural deficits and heightened risk of intracranial complications. The findings underscore the value of early radiologic classification, particularly using high-resolution imaging, to inform risk stratification and guide tailored management. Surgical intervention, when appropriately indicated, yields meaningful auditory improvement, although vestibular recovery appears to rely more heavily on structured rehabilitation than on operative measures. These findings directly inform clinical practice: (1) OCV fractures warrant early counseling regarding permanent hearing loss and cochlear implant candidacy, (2) surgical intervention provides measurable audiometric benefit (7.6 dB improvement) justifying operative risk, and (3) structured vestibular rehabilitation should be prioritized given demonstrated acceleration of recovery. Cost-effectiveness analyses suggest early intervention protocols may reduce long-term disability burden.

## Data Availability

The raw data supporting the conclusions of this article will be made available by the authors, without undue reservation.
